# Effect of replacing concentrate feed with leaves of Oldman saltbush (*Atriplex nummularia*) on feed intake, weight gain, and carcass parameters of highland sheep fed on wheat straw in northern Ethiopia

**DOI:** 10.1007/s11250-018-1577-8

**Published:** 2018-03-25

**Authors:** Kidane Hintsa, Amanuel Berhe, Mulubrhan Balehegn, Kidane Berhe

**Affiliations:** 1 College of Dry land Agriculture and Natural Resources, Department of Animal, Rangeland and Wildlife Sciences, Mekelle University, P.o.Box 231, Mekelle, Ethiopia; 2 Sustainable Land Management (SLM), Gesellschaft für Internationale Zusammenarbeit (GIZ), Mekelle, Tigray Ethiopia

**Keywords:** *Atriplex nummularia*, Body weight gain, Carcass parameter, Ethiopia, Feed intake, Sheep

## Abstract

*Atriplex nummularia* (oldman saltbush) is widely planted on salt-affected land to provide a vegetative cover, which can be used as an alternative feed resource. The study was conducted to evaluate the replacement of commercial concentrate with oldman saltbush (SB) leaf on the feed intake, live weight gain, and carcass parameters of local sheep. Twenty-five sheep with an age of 6 months and weighting 14.60 ± 2.47 kg were used in randomized complete block design and distributed into five equal groups, with five animals each. The selected sheep were fed with the dietary treatments for 2 weeks for adaptation and continued until the end of the study, which lasted for 90 days. In the control treatment (SB-0), sheep were fed 100% concentrate mix, while in SB-5, SB-10, SB-15, and SB-20, 5, 10, 15, and 20% of the concentrate mix was replaced by SB, respectively. Wheat straw and water were given at ad libitum throughout the experiment period. Data on feed intake and live weight gain were recorded daily and weekly, respectively, for 90 days. Three animals from each treatment were slaughtered for carcass analysis. *Atriplex nummularia* leaf (Oldman saltbush) contained 92.02% dry matter (DM), 21.99% crude protein (CP), 24.77% ash, 58.27% neutral detergent fiber (NDF), and 26.56% acid detergent fiber (ADF). Dry matter intake, live weight gain, and carcass parameter were not statistically different among the treatments. Result indicated that concentrate feed can be replaced with *Atriplex nummularia* leaf up to 20% in sheep diet without negative effect on growth performance and carcass characteristics.

## Introduction

The production and productivity of the livestock sector in Ethiopia is very limited even though the country has huge potential. Ethiopia has an estimated livestock population of 59.5 million cattle, 30.7 million sheep, 30.2 million goats, and 56.53 million poultry (CSA 2017), assumed to be the largest livestock number in Africa. Sintayehu et al. (2008) reported that the livestock sector is less productive and its contribution to the national economy is low. This low livestock production and productivity might be due to mainly poor feed quality and quantity among other constraints (Zegeye 2003). Likewise, McDonald et al. (2002) reported that the feed by-products like straw and others are low nutritional value and less digestible. High fibrous feeds reduce animals’ feed intake. Previously, Van Soest (1994) reported that the Ethiopian dry forages and roughages contain less than 7% of crude protein and this amount of protein is low to meet the microbial requirement. When animals are fed these feeds alone, they cannot even satisfy their maintenance requirement (FAO 1999). Moreover, the rapidly increasing human population and its demand for food production have caused the conversion of rangelands and pasturelands into cultivation land; resulting livestock is kept in areas with low potential lands for crop production (Alemayehu 2005). The change of rangelands and pasturelands into cultivation land is anticipated to proceed at an accelerating pace with population growth. Moreover, with the present trend of fluctuating feed cost and global inflation, livestock production is increasingly constrained by feed scarcity and the high cost of feeds (Ayantunde et al. 2005).

Alternatively, fodder shrubs such as *Atriplex nummularia* would reduce feed shortage while improving degraded grazing lands. The plant grows on wide array of area including saline area and is used to mitigate feed shortages within grazing systems (Le Houerou 1992). It is appreciated for its high crude protein content (Ben Salem et al. 2002) and low secondary metabolites such as tannins and saponins (Ahmed et al. 2015). These resulted improvements of ruminants’ performance either grazed or were stall-fed on Atriplex (Ben Salem et al. 2010). *Atriplex nummularia* was recently introduced to Ethiopia and is being well grown in different parts of the country and adapted in Tigray Region in northern Ethiopia. However, the forage value of this plant has not been considered by farmers. Moreover, the effect of feeding *A*. *nummularia* on the productivity of animals and its optimal level of replacement for commercial concentrate feeds have not yet been identified. This study was therefore conducted to investigate the optimal replacement level of expensive commercial concentrate feeds with dried leaves of *Atriplex nummularia* leaf on the productive performance and carcass characteristics of highland sheep in northern Ethiopia.

## Materials and methods

### Description of study area

The experiment was conducted in Mekelle University at small ruminant farm unit. The university is located at latitude and longitude of 13° 27′ N and 039° 01′ E, respectively. Altitude of the area ranges between 2000 and 2200 m.a.s.l. with semi-arid climate. The annual rainfall ranges from 500 to 700 mm. The mean annual temperature ranges from 20 to 30 °C. Oldman saltbush leaf for the feeding trial was harvested from Kola Temben district, Central Tigray. The district is located at latitude and longitude of 13° 37′ 23″ North and 38° 00′ 05″ East, respectively. The district has 137,755.7 m^2^ total area coverage. The altitude of the district ranges from 913 to 2553 m.a.s.l. with agro-climatic zones of lowland (81%), midland (18%), and highland (1%). The district is characterized by sandy, clay, and silt soil types. Mixed crop-livestock farming is a typical farming system in the district. Pasture and crop residue mainly the stalk of maize and sorghum and teff straw are the main livestock feed resources of the area.

### Experimental diet preparation

Oldman saltbush leaf for the feeding trial was collected from Kola Temben district in Tigray Regional State where the area has good potential. Upon use, leaves were allowed to air-dry and were grounded in a miller before mixing the diets, to ensure thoroughness during the mixing process. The leaves grounded using hummer mill were made to pass through 25-mm sieve following the procedure outlined. Concentrate feed were purchased from Mekelle city market. Before mixing the diets, Oldman saltbush leaves were dried under open shade and this made ease for grinding process. Experimental diets were mixed every 10 days.

The experimental feed consisted of concentrates mixture composed of maize 35%, cotton seed cake 32%, wheat bran 30%, salt 1%, and mineral premix 2%. Animals were given 2.5% of their live body weight on daily basis.

### Animals, experimental design, and treatments

Total of 25 sheep of similar age (6 months), sex (male), and weight 14.60 ± 2.47 kg were used in randomized complete block design (RCBD) experiment with five dietary treatments of each five animals. The experimental animals (highland breeds of sheep) were purchased from Atsibi wemberta district, eastern Tigray, Ethiopia. A concentrate mixture of (maize 35%, wheat bran 30%, cotton seed cake 32%, common salt 1%, mineral mixture 2%) was given at 2.5% of their body weight for the control group. For the treatment groups SB-5, SB-10, SB-15, and SB-20, 5, 10, 15, and 20% of the concentrate were replaced by equal amount of dried *Oldman saltbush* leaf, respectively. Wheat straw was the basal diet and was provided ad libitum together with water. Therefore, sheep were blocked based on their initial body weight into five blocks of five animals, and five dietary treatments were randomly assigned to animals in the block. Animals were sprayed with diazinol and dewormed and dosed with Ivermectin injection for ecto- and endoparasite control. Besides, animals were given vaccine against pasteurellosis, anthrax, and sheep pox.

### Feed intake

This experiment was conducted for 104 days with the first 2 weeks as an adaptation period, where experimental animals were fed with their specific treatments throughout the adaptation period. The feed offered and refused corresponding to each treatment and animals were recorded daily throughout experimental period and daily feed intake (DFI) was calculated as the difference between offer and refusal. Samples of daily feed offered and refused were pooled over experimental period for each treatment group and were stored in plastic bags. Sub-samples of feed offered and refused were taken for each treatment group and were dried at 65 °C for 4 h in an oven pending for chemical analysis. The DM and nutrient intakes were determined as a difference between the amounts offered and refused for each feed and treatment.

### Live weight changes feed conversion efficiency

Body weight of animals was measured at weekly interval for 90 days. Sheep were weighed during morning hours before feeding and watering. The average daily weight gains (ADG) was calculated on a weekly basis as the difference between final live weight and initial live weight divided by the number of days. Feed conversion efficiency (FCE) was measured as proportion of average daily weight gain (ADG) to daily feed intake (DFI).

### Carcass parameters

At the end of the experiment, 15 sheep (three per treatment) were randomly selected for carcass analysis. These sample sheep were fasted overnight and then their fasted live weight (FLW) were recorded. During slaughter, the weights of non-carcass components and the hot carcass were recorded. Non-carcass components such as blood, head + limb, skin + tail, testicles + penis, thoracic organs (heart, diaphragm, lungs, and trachea), and viscera (liver, spleen + kidneys) were weighed and recorded. The weight of the digestive tract was calculated as the difference between empty digestive tract and the weight of full at fasting. Empty body weight (EBW) was calculated by subtracting the digestive tract from the FLW. The dressing percentage was calculated as proportion of the hot carcass weight to the percentage of FLW.

### Chemical analysis

Dry matter (DM), ash, crude protein (CP), acid detergent fiber (ADF), neutral detergent fiber (NDF), and acid detergent lignin (ADL) of experimental feeds (concentrate feed, Oldman saltbush leaves, and samples feed offered) were analyzed using standard procedures of AOAC (AOAC 2000).

### Statistical analysis

Data collected were subjected to statistical analysis of variance (ANOVA) using the general linear model (GLM) in SPSS-2011 version 20 statistical software that was used to analyze the data.

## Result and discussion

### Chemical composition of treatment feeds

Chemical compositions of feed ingredients and treatments used in this experiment are presented in Tables 1 and 2, respectively. Wheat straw (WS) had a CP content below the minimum microbial requirement (7%) in feeds to support acceptable ruminal microbial activity and the maintenance requirement of CP for the host ruminant (McDonald et al. 2002), which necessitates supplementation. High contents of NDF and ADF reduce feed intake and digestibility (Van Soest 1994). The NDF and ADF contents of WS found in this experiment were lower as compared to the results 7.2 and 3.84% reported by Temesgen and Yayneshet (2013). The CP value of WS of this study was higher than the values 2.5 and 3% reported by Temesgen and Yayneshet (2013) and Gebremeskel and Kefelegn (2011), respectively.

**Table 1:** Chemical composition of experimental feeds (percentages)

Feed	Nutrient				
	DM	CP	Ash	NDF	ADF
Cottonseed cake	97.5	35.5	4.2	54.4	11.1
Wheat bran	83.1	15.9	3.9	30.2	8.4
Maize	91.8	8.7	1.1	10.8	2.9
Oldman saltbush	92	21.9	24.8	58.3	28.6
Wheat straw	97.5	4.7	5.5	0.6	2.8

*DM* dry matter, *CP* crude protein, *NDF* neutral detergent fiber, *ADF* acid detergent fiber, *ADL* acid detergent lignin

**Table 2:** Chemical composition of treatments

Composition	Treatments				
	SB-0	SB-5	SB-10	SB-15	SB-20
DM (%)	90.2	91.7	91.9	90.3	89.9
CP (%)	15.9	15.3	15.8	15.7	16.4
ADF (%)	12.5	11.7	13.3	13.9	13.7
NDF (%)	23.2	21.2	24.2	25.2	25.3
Ash (%)	3.6	4.1	6.4	6.7	7.3
EE (%)	4.6	4.8	4.9	4.8	4.6

*DM* dry matter, *CP* crude protein, *NDF* neutral detergent fiber, *ADF* acid detergent fiber, *ADL* acid detergent lignin, *SB* saltbush. *SB-0* control (maize 35%, wheat bran 30%, cotton seed cake 32%, common salt 1%, mineral mixture 2%), *SB-5* (saltbush 5% replaced to concentrate feed), *SB-10* (saltbush 10% replaced to concentrate feed), *SB-15* (saltbush 15% replaced to concentrate feed), *SB-20* (saltbush 20% replaced to concentrate feed). Wheat straw was ad libitum in all the treatments

The CP content of Oldman saltbush (SB) was high compared with that of maize and wheat bran. The CP content of Oldman saltbush used in the present study (21.99%) was higher than the values (14–19) reported by Norman et al. (2004) and lower than the values of 25.2% reported by Khalil et al. (1986), but similar with the value (21.4%) reported by Wehren (1976). The NDF (32.68%) of SB obtained in the present study is lower than with the values of 60% reported by El Shaer and Zahran (2002). The result of the present study indicated the nutritional content of Oldman saltbush leaf is comparable to that of commercial concentrates. The crude protein content of maize in the present study is lower than the values reported by Kidane et al. (2015). The crude protein content of cottonseed cake (35.5%) in the present study is higher than the values reported by Ndemanisho et al. (1998), while the NDF and ADF values is lower than the values reported by Ndemanisho et al. (1998). The CP of wheat bran (WB) content in current study is higher than the value reported by Temesgen and Yayneshet (2013), but it is lower than the value reported by Kidane et al. 2015. The NDF and ADF values of wheat bran in the present study was lower than the values 46 and 34.2% reported by Temesgen and Yayneshet (2013) and Gebremeskel and Kefelegn (2011), respectively. The variation among chemical contents might be due to variations in the age of the plants at harvest, the processing methods used, and leaf to petiole ratio in the sample. The DM, CP, NDF, and ash of SB-20 diets were higher than those of SB-0, SB-5, SB-10, and SB-15. However, the ADF content of SB-20 is lower than that of SB-15 but higher than those of SB-0, SB-5, and SB-10.

### Dry matter and nutrient intake

There was no significant difference (*P* > 0.05) intake of the experimental feed between treatments and within replication (Table 3).

**Table 3:** Daily feed intake of each treatment

Feed (g/head/day)	Treatment				
	SB-0	SB-5	SB-10	SB-15	SB-20
Straw intake	250 ± 4.69	248.40 ± 2.6	251.60 ± 7.3	254.39 ± 13.4	247.63 ± 10.2
Concentrate intake	345.53 ± 60.7	344.70 ± 49.2	344.80 ± 49.3	343.97 ± 44.1	342.19 ± 47.6
Total intake	595.50 ± 60.7	592.97 ± 48.1	596.38 ± 51.5	598.88 ± 43.5	589.88 ± 52.3

*SB-0* control (maize 35%, wheat bran 30%, cotton seed cake 32%, common salt 1%, mineral mixture 2%), *SB-5* (saltbush 5% replaced to concentrate feed), *SB-10* (saltbush 10% replaced to concentrate feed), *SB-15* (saltbush 15% replaced to concentrate feed), *SB-20* (saltbush 20% replaced to concentrate feed). Wheat straw was ad libitum in all the treatments

Similarly, Obeidat et al. (2016) did not observe significant differences in the dry matter intake animals fed diets containing different proportions of Oldman saltbush. However, saltbush-supplemented animals increased their DMI significantly as compared to non-supplemented animals (Abu-Zanat and Tabbaa 2006).

### Live weight changes and feed conversion efficiency

There was no significant difference (*P* > 0.05) on average daily live weight gain of sheep between treatments (Table 4). Similarly, the treatments had no significant effect on the final BW of ewes supplemented with saltbush (Abu-Zanat and Tabbaa 2006), and animals offered straw while grazing Oldman saltbush in the field has not demonstrated higher productivity than animals without supplements (Franklin-McEvoy et al. 2007; Norman et al. 2008). However, daily live weight gain increased in the animals supplemented with saltbush than the control groups across the entire experiment period (Ahmed et al. 2015).

**Table 4:** Initial and final live body weight of animals and their feed conversion efficiency

Parameter	Treatment				
	SB-0	SB-5	SB-10	SB-15	SB-20
Initial body weight (kg)	15.62 ± 2.8	15.30 ± 2.9	15 ± 2.8	13.83 ± 1.8	13.60 ± 2.4
Final body weight (kg)	19.35 ± 3.9	18.84 ± 3.1	18.92 ± 2.8	17.16 ± 2.2	17.04 ± 2.2
Average weight gain (g/head/day)	41.38 ± 14.3	39.33 ± 15.2	43.55 ± 1.9	37.03 ± 15.7	38.22 ± 9.4
Feed conversion efficiency (%)	15.32 ± 3.9	17.67 ± 8.4	13.73 ± 1.6	19.35 ± 10.03	16.66 ± 6.6

*SB-0* control (maize 35%, wheat bran 30%, cotton seed cake 32%, common salt 1%, mineral mixture 2%), *SB-5* (saltbush 5% replaced to concentrate feed), *SB-10* (saltbush 10% replaced to concentrate feed), *SB-15* (saltbush 15% replaced to concentrate feed), *SB-20* (saltbush 20% replaced to concentrate feed). Wheat straw was ad libitum in all the treatments

There was no significant difference (*P* > 0.05) on feed conversion efficiency of sheep among treatments (Table 4). Recently, Obeidat et al. (2016) also reported that the feed conversion of experimental animals fed diets containing different proportions of Atriplex halimus L was not altered. Result of the present study indicated that the replacement of commercial concentrates with Oldman saltbush did not show difference on FCE of different treatments.

### Carcass parameters

The average values of hot carcass weight and edible carcass offal were not significantly different among the different treatments (Table 5). In agreement with the present study, Obeidat et al. (2016) did not observe any difference in carcass components due to the replacement of saltbush in various proportions. Therefore, the results indicated the possibility of replacing nutrient-rich concentrate feeds by saltbush up to 20% in sheep diet without affecting carcass characteristics and carcass cut proportions.

**Table 5:** Edible carcass components of slaughtered animal

Parameter (kg)	Treatment				
	SB-0	SB-5	SB-10	SB-15	SB-20
Fasted body weight	17.73 ± 5.9	18.06 ± 2.8	17.07 ± 3	16.23 ± 2.6	17.25 ± 1.8
Empty body weight	14.60 ± 3.9	15.21 ± 2.8	14.23 ± 2.5	13.26 ± 1.8	14.87 ± 1.5
Hot carcass weight	7.03 ± 2.4	7.25 ± 1.5	6.62 ± 1.2	6.23 ± 1.3	6.90 ± 1.3
Dressing percentage	39.64 ± 0.6	39.92 ± 2.0	38.75 ± 1.1	38.21 ± 2.2	38.83 ± 3.3
Total edible portion	5.36 ± 1.4	6.01 ± 2.4	5.41 ± 1.8	4.66 ± 0.6	6.22 ± 1.02
Total saleable portion	3.85 ± 0.9	3.50 ± 0.8	3.20 ± 0.8	3.21 ± 0.7	3.55 ± 0.6
Neck	0.46 ± 0.2	0.38 ± 0.1	0.32 ± 0.1	0.36 ± 0.1	0.47 ± 0.03
Proximal thoracic limb	0.80 ± 0.2	0.75 ± 0.2	0.72 ± 0.1	0.70 ± 0.2	0.70 ± 0.1
Steak + brisket	0.83 ± 0.1	0.66 ± 0.2	0.66 ± 0.2	0.71 ± 0.1	0.77 ± 0.2
Lumbar + abdominal region	0.81 ± 0.2	0.75 ± 0.2	0.65 ± 0.2	0.70 ± 0.3	0.65 ± 0.1
Proximal pelvic limb	0.91 ± 0.2	0.95 ± 0.1	0.83 ± 0.3	0.73 ± 0.2	0.95 ± 0.2

*SB-0* control (maize 35%, wheat bran 30%, cotton seed cake 32%, common salt 1%, mineral mixture 2%), *SB-5* (saltbush 5% replaced to concentrate feed), *SB-10* (saltbush 10% replaced to concentrate feed), *SB-15* (saltbush 15% replaced to concentrate feed), *SB-20* (saltbush 20% replaced to concentrate feed). Wheat straw was ad libitum in all the treatments

There was no significant difference in the weight of different non-edible carcass components among the different treatments (Table 6). In agreement with the present study, Obeidat et al. (2016) reported that the values of non-edible carcass components did not show significant difference among the treatments. Therefore, the results indicated the possibility of replacing nutrient-rich concentrate feeds with Oldman saltbush leaf up to 20% in sheep diet without negative effect on carcass characteristics and non-carcass components.

**Table 6:** Non-edible carcass components of the slaughtered animals

Parameter (kg)	Treatment				
	SB-0	SB-5	SB-10	SB-15	SB-20
Head and limb	2.13 ± 0.4	1.90 ± 0.2	2.17 ± 0.3	1.93 ± 0.3	2.10
Skin and tail	1.83 ± 0.5	1.76 ± 0.2	2.20 ± 0.4	1.86 ± 0.3	2.05 ± 0.1
Testicle and penis	0.30 ± 0.1	0.30 ± 0.1	0.37 ± 0.1	0.23 ± 0.1	0.25 ± 0.1
Gut content	3.13 ± 2.1	2.43 ± 0.9	3.15 ± 0.9	2.96 ± 0.9	2.37 ± 0.2
Empty digestive track	1.13 ± 0.2	1.13 ± 0.1	1.10 ± 0.2	1.03 ± 0.1	1.17 ± 0.1
Diaphragm, lung, trachea and heart	0.53 ± 0.2	0.46 ± 0.1	0.45 ± 0.1	0.43 ± 0.1	0.50
Kidney and spleen	0.16 ± 0.1	0.13 ± 0.1	0.17 ± 0.1	0.13 ± 0.1	0.20
Liver	0.16 ± 0.1	0.26 ± 0.1	0.25 ± 0.1	0.13 ± 0.1	0.20

*SB-0* control (maize 35%, wheat bran 30%, cotton seed cake 32%, common salt 1%, mineral mixture 2%), *SB-5* (saltbush 5% replaced to concentrate feed), *SB-10* (saltbush 10% replaced to concentrate feed), *SB-15* (saltbush 15% replaced to concentrate feed), *SB-20* (saltbush 20% replaced to concentrate feed). Wheat straw was ad libitum in all the treatments

## Conclusion

Result indicated the potential utilization of *Atriplex nummularia* leaf as a replacement feed for concentrate feed without negative effect on growth performance and carcass characteristics. Therefore, the use of this plant as an alternative feed resource for livestock may mitigate the shortage of feed sources for small holder farmers.
